# Cross Species Analysis and Comparison of Tumors in Dogs and Cats, by Age, Sex, Topography and Main Morphologies. Data from Vet-OncoNet

**DOI:** 10.3390/vetsci9040167

**Published:** 2022-03-31

**Authors:** Katia Pinello, Isabel Pires, Ana Filipa Castro, Paulo Tiago Carvalho, Andreia Santos, Augusto de Matos, Felisbina Queiroga, Ana Canadas-Sousa, Patrícia Dias-Pereira, José Catarino, Pedro Faísca, Sandra Branco, Cristiana Lopes, Filipa Marcos, Maria C. Peleteiro, Hugo Pissarra, Pedro Ruivo, Rui Magalhães, Milton Severo, João Niza-Ribeiro

**Affiliations:** 1 Departamento de Estudo de Populações, Vet-OncoNet, ICBAS, Instituto de Ciências Biomédicas Abel Salazar, Universidade do Porto, 4050-313 Porto, Portugal; up201505657@edu.icbas.up.pt (A.F.C.); up200703166@edu.icbas.up.pt (P.T.C.); rmag@icbas.up.pt (R.M.); jjribeiro@icbas.up.pt (J.N.-R.); 2 EPIUnit-Instituto de Saúde Pública, ISPUP, Universidade do Porto, 4050-600 Porto, Portugal; msevero@icbas.up.pt; 3 Laboratório para a Investigação Integrativa e Translacional em Saúde Populacional (ITR), 4050-600 Porto, Portugal; 4 Departamento de Ciências Veterinárias, Universidade Trás-os-Montes e Alto Douro (UTAD), 5000-801 Vila Real, Portugal; ipires@utad.pt (I.P.); fqueirog@utad.pt (F.Q.); 5 CECAV-Centro de Ciência Animal e Veterinária, Universidade de Trás-os-Montes e Alto Douro, 5001-801 Vila Real, Portugal; 6 Departamento de Clínicas Veterinárias, ICBAS, Instituto de Ciências Biomédicas de Abel Salazar, Universidade do Porto, 4050-313 Porto, Portugal; aasantos@icbas.up.pt (A.S.); ajmatos@icbas.up.pt (A.d.M.); 7 CECA-ICETA-Centro de Estudos de Ciência Animal, Instituto de Ciências, Tecnologias e Agroambiente, Universidade do Porto, 4050-083 Porto, Portugal; 8 Departamento de Patologia e Imunologia Molecular, ICBAS, Instituto de Ciências Biomédicas Abel Salazar, Universidade do Porto, 4050-313 Porto, Portugal; amcanadas@icbas.up.pt (A.C.-S.); pdpereira@icbas.up.pt (P.D.-P.); 9 DNAtech, Veterinary Laboratory, 1649-038 Lisboa, Portugal; p5663@ulusofona.pt (J.C.); pedrofaisca@ulusofona.pt (P.F.); 10 Faculdade de Medicina Veterinária, Universidade Lusófona de Humanidades e Tecnologia, 1749-024 Lisboa, Portugal; 11 CBIOS-Research Center for Biosciences and Health Technologies, Universidade Lusófona de Humanidades e Tecnologia, 1749-024 Lisboa, Portugal; 12 Instituto Mediterrâneo para a Agricultura, Ambiente e Desenvolvimento, MED, Universidade de Évora, 7006-554 Évora, Portugal; smbb@uevora.pt; 13 VetPat, Veterinary Pathology Laboratory, 1500-580 Lisboa, Portugal; cristianalopes25@hotmail.com (C.L.); marcos.filipa@gmail.com (F.M.); mcpeleteiro@gmail.com (M.C.P.); hpissarra@fmv.ulisboa.pt (H.P.); prruivo@ucdavis.edu (P.R.)

**Keywords:** cancer, cat, dog, epidemiology, oncology, veterinary

## Abstract

The animal cancer burden is essential for the translational value of companion animals in comparative oncology. The present work aims to describe, analyze, and compare frequencies and associations of tumors in dogs and cats based on the Animal Cancer Registry created by Vet-OncoNet. With 9079 registries, regarding 2019 and 2020, 81% (*n* = 7355) belonged to dogs. In comparison, cats have a general one-year right advance in the mean age of cancer diagnosis compared to dogs. The multivariate topography group analysis shows a distinct pattern between the two species: dogs have higher odds of cancer in the genito-urinary system, spleen, soft tissue tumors and skin, while cats show higher odds for tumors in the eyes, digestive organs, nasal cavity, lymph nodes, bones and mammary glands. Regarding morphologies, dogs are overrepresented in mast cell tumors (MCT), melanomas, and hemangiosarcomas. While cats are overrepresented in fibrosarcomas, lymphomas (T and B-cell), in malignant mammary tumors, and squamous cell carcinoma (SCC). Females have greater odds only in the mammary gland, with males having greater odds in six of twelve topographies. This study is the first outcome of continuous animal cancer registration studies in Portugal.

## 1. Introduction

Companion animals live in more than 88 million households in Europe (38% of all) with around 90 million dogs and 110 million cats [1]. Natural occurring diseases in companion animals, like cancer, are often similar and sometimes identical to human diseases, in terms of etiology, progression and treatment response [2,3].

Dogs are considered a useful, attractive, and complementary model of human cancer as sharing human physical, chemical environments, and approximately “650 Mb of ancestral genetic sequence” [3,4,5]. From a histological perspective, numerous cancer types, e.g., osteosarcomas, melanomas, non-Hodgkin lymphomas, bladder cancer, and mammary carcinomas are quite similar in dogs and humans [6,7,8,9,10,11].

Domestic cats offer the same potential as dogs as a model, and may be even a better fit for some specific type of tumors, although they are not so often utilized to the same degree as dogs in the One Medicine approach to cancer [12]. Feline oral squamous cell carcinoma (SCC), which shares both clinical and molecular features with human head and neck cancer [13], and feline mammary tumors, sharing the ‘triple-negative’ phenotype with breast cancer in humans [12,14] offer an enriched population model for evaluating new potential targets, treatments and environmental risk factors for cancer research [12,13,14].

Only with an accurate animal cancer surveillance it is possible to produce important scientific evidence for the potential translational key role of companion animals in comparative studies with humans [5,11]. This important epidemiologic tool makes it possible to generate a hypothesis that enables the design of more precise analytic studies to identify causal associations between exposures and cancer risks [15]. Animal cancer data bases are also essential tools for cancer prevention and control in animals and humans [15].

Aware of its importance, Vet-OncoNet, the Veterinary Oncology Network [16] has, included in its mission, the construction and maintenance of an Animal Cancer Registry (ACR) representative of Portuguese reality. The system includes the collection, treatment and reporting of data on tumors in companion animals to produce scientific evidence, that contributes to improve the knowledge in comparative oncology [4].

A study comparing the occurrence of tumor topographies and morphologies in cats and dogs can provide an important insight to similarities and differences in tumor occurrence, contributing to generate evidence and hypothesis to support and guide clinical and comparative research questions.

The present work is a cross-sectional epidemiological study based on the ACR established by Vet-OncoNet [16], in Portugal, and intends to compare tumor occurrence in dogs and cats. To achieve this objective, frequencies and associations of tumor topographies and main morphologies in dogs and cats are described, analyzed and compared, considering age and sex, as reported by several veterinary diagnostic laboratories in Portugal.

## 2. Materials and Methods

### 2.1. Data Origin and Structure

Data of confirmed animal cancer diagnosis referred to January 2019 and December 2020 that were sent to Vet-OncoNet by five veterinary laboratory partners (VLP) in Portugal: DNATECH, VetPat^®^, Laboratory of Pathological Anatomy from the Faculty of Veterinary Medicine, University of Lisbon, the Laboratory of Veterinary Pathology, University of Porto and the Laboratory of Veterinary Pathology, University of Évora. The data provided includes date of diagnostic, species, age, sex, postal code, diagnosis (morphology), as well as tumor grade and localization (topography).

### 2.2. Data Preparation

When entering in the system, the data undergo a first stage of cleaning and treatment comprising editing, validation, standardization of the terms coming from the different pathologists, and classification. When necessary, clarification of the information received was required from the pathologist. All the tumor cases with diagnosis were included in the database; however, some records lacked all parameters due to incomplete information submitted by the clinical veterinarian to the VLP.

Each record was classified accordingly to the anatomical localization (topography) and histological type (morphology) using the Vet-ICD-O-canine-1 classification system [17]. This system, developed by the Global Initiative on Veterinary Cancer Surveillance (GIVCS) in close collaboration with the International Agency for Research in Cancer (IARC) [18], is the canine counterpart of the human classification, ICD-O-3.2 (International Classification of Diseases–Oncology, version 3.2). Tumors’ topographies were grouped in 14 “topography groups”, accordingly to the 23rd volume of the Northern Regional Cancer Registry (RORENO) [19], as listed on Table 1, for the sake of future comparability with the human registry. The term NOS (not otherwise specified) was adopted when specific information (topography or morphology) was not sufficiently precise for classification. The connective, subcutaneous and other soft tissues tumors group was not split in sub-locations due to the lack of information in many of the records.

Due to the high diversity of tumors present, the morphological analysis required decisions regarding grouping or suppression as follows. Only the most relevant morphology entities were addressed individually, and for morphologies with a low number it was decided to group them. For convenience of the analysis, mammary tumors were divided in two groups accordingly the diagnosis: benign or malignant tumors; and in mast cell tumors (MCT), cutaneous and subcutaneous, independently of the grade, were grouped together. Regarding the lymphomas group, epitheliotropic and non-epitheliotropic were grouped under the name “Cutaneous lymphomas” and when phenotype was provided B- and T-cell were discriminated.

### 2.3. Data Analysis

Descriptive analysis was performed using counts, median and percentages for categorical variables, and mean and standard deviation (SD) for continuous variables. The Z-test was used to assess differences between proportions and, for continuous variables, the Student’s t-test and ANOVA were used. Kolmogorov–Smirnov test (K–S test) was performed to test for normality and compare the cumulative distribution incidence age of the tumors among species and sex.

Sex proportion ratios (PR^f/m^) were calculated for tumor topographies and morphologies as the ratio between the proportions of a specific tumor in females over the proportion of the same tumor in males. PR are presented with the respective 95% confidence intervals (95%CI), calculated using the first order Taylor series approximation [20].

Crude odds ratios (OR) were calculated to find over representation of species (dog/cat) in tumor’s topography and morphology. Fisher’s exact test was used to assess *p*-values for crude OR. Binary logistic regression models were used to calculate adjusted OR having as independent variables species, sex and age (one year each) and as dependent variables topography groups or morphologies.

In the results section, every time differences are mentioned, a significance level of 5% or lower is implicit, unless stated otherwise. Data were analyzed using Statistical Package for Social Sciences (SPSS) 26.0 (IBM Corp, Armonk, NY, USA) and R version 4.0.3.

## 3. Results

Vet-OncoNet received 9182 registries with more than 98% (*n* = 9079) concerning dogs (*n* = 7355, 81%) and cats (*n* = 1724, 19%). Other species were rodents (*n* = 27, 0.30%), lagomorphs (*n* = 24, 0.25%), horses (*n* = 22, 0.24%) and others (reptiles, birds, ferrets) with less than 0.1%. Table 2 shows differences between sex within and among species. Dogs were four times more represented in the sample than cats, and females, from both species, represent almost 60% of the overall cases (Table 2).

### 3.1. Age’s Analysis

**Figure 1:**
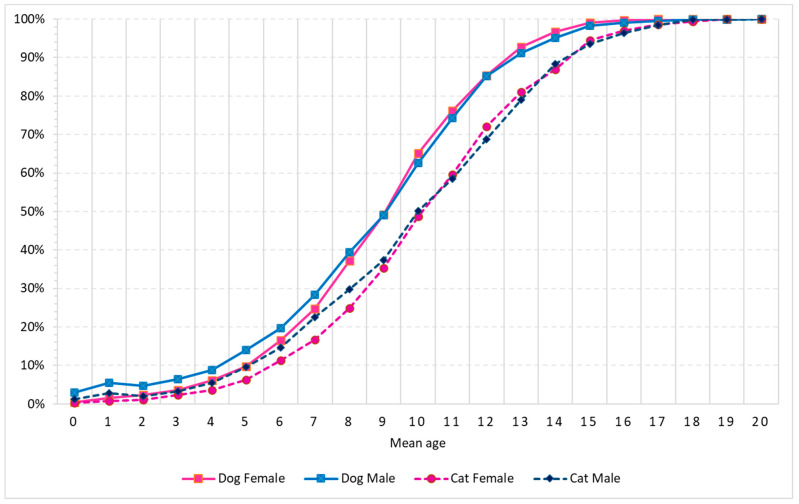
Cumulative case proportion of mean age of incidence by species and sex (in years).

The number of age records were 7245 dogs and 1689 cats (Table 2). Age of the animals in the registries ranged from three months old for dogs, and six months old for cats up to 20 years old (y.o) in both species (Table 2 and Figure 1) with an overall mean age of incidence of 9.5 y.o (SD 3.2). Table 2 shows that dogs’ mean age is lower than cats’: 9.3 and 10.5 years old, respectively. The difference between species was kept among sex, although within species, the sex mean age does not differ. Figure S1 shows that the highest number of cases are seen at 10 years in dogs (*n* = 989, 14.9%) and cats (*n* = 201; 13.2%). There is a second age peak for dogs at eight years old (*n* = 784; 11.9%) and for cats at twelve years old (*n* = 178; 11.6%) (Figure S1).

Statistical analysis of differences among species and sex, using the Kolgomorov–Smirnov test (Figure 1), showed significant differences on the age of cumulative incidence among dogs and cats and among sex within both species, for certain parts of the curves, which are detailed as follows. Within dogs: males have higher incidence than females up to seven years old, although the differences disappear from eight years onwards. Within cats, no difference was found as age accumulate. Among species, male dogs show higher cumulative incidence up to 14 or 13 years old than female or male cats, respectively. Female dogs and male cats have comparable age cumulative incidence before eight- and after fourteen-years old. However, between eight- and fourteen-years old female dogs have higher cumulative incidence than male cats. Female dogs have earlier incidence than female cats up to fourteen years old.

### 3.2. Topographies’ Analysis

The analysis of the distribution of tumors per topography group listed on Table 3 shows that skin (*n* = 4213, 46.4%) and mammary gland (*n* = 2313, 25.4%) are by far the most frequent affected systems. For dogs, the third most represented system is the genito-urinary (*n* = 555, 7.7%) and for cats is the digestive organs (*n* = 138, 8.2%).

The univariate analysis (Table 3) shows that dogs are more likely (*p* < 0.05) than cats to present tumors in genito-urinary organs, soft tissues, spleen, skin and oral cavity. Cats are more likely to have tumors in the digestive organs, respiratory, mammary gland, eyes and lymph nodes. There is no species overrepresentation in the endocrine glands and bone/joints.

#### 3.2.1. Oral and Pharyngeal Cavity

**Figure 2:**
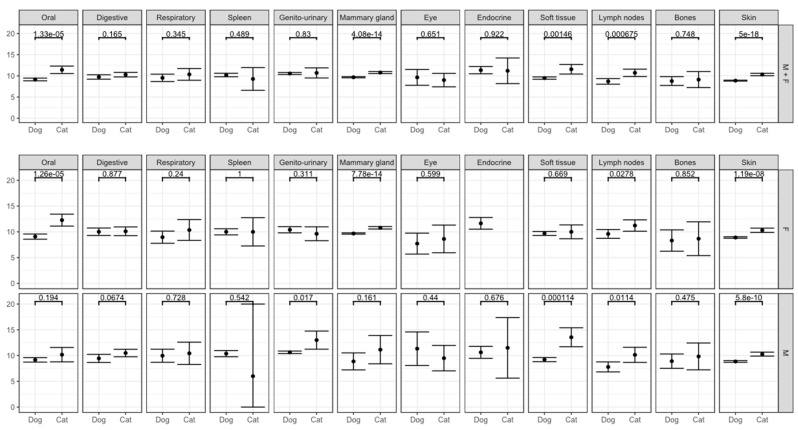
Mean age distribution of the topography groups by species and sex with the respect *p*-value.

Table 3 shows that tumors from this topography group are 1.3-fold overrepresented in dogs and that gum tumors are the most frequent in both species. Cats have almost three times the odds of tumors in the tongue compared to dogs. Male dogs show a higher proportion in all topography items with the exception to the tongue. Regarding age of incidence (Figure 2 and Table S1), dogs present a lower age than cats and female cats have higher mean age than males.

#### 3.2.2. Peritoneum and Digestive Organs

The intestinal tract is the main localization within this topography group for either species and mostly in cats (Table 3). The intestinal tract and stomach are overrepresented in cats when compared to dogs. Concerning sex, males from both species have a higher proportion of intestinal tract tumors than females (Table 3). There is no difference in the mean age incidence, neither by species, nor by sex (Figure 2 and Table S1).

#### 3.2.3. Respiratory System, Intrathoracic Organs

The nasal cavity is the area where tumors are most frequently found in both species within this group. Cats have three times higher odds than dogs. Male dogs show a higher proportion than females, not seen in cats (Table 3). No significant differences in the mean age are observed between species or sex (Figure 2 and Table S1).

#### 3.2.4. Hematopoietic System and Endothelial Reticulum

Tumors in the spleen are 2.5-fold overrepresented in dogs than in cats without sex predisposition in both species (Table 3). No differences in mean age of incidence were found by species neither sex (Figure 2 and Table S1).

#### 3.2.5. Genito-Urinary Organs

In an overall analysis, dogs show five-fold higher odds of having tumors in this topography group than cats (Table 3), with the male genital organs (testicles and scrotum locations) being responsible for the differences observed. Cats almost do not present tumors in genital organs with the exception for tumors in the uterus where female cats have three times the odds of dogs (Table 3). Regarding only urinary organs, there are no differences by species or sex. There is no difference in mean age by species and female cats showed a lower mean age of incidence than male cats (Figure 2 and Table S1).

#### 3.2.6. Mammary Gland

Cats are two-fold overrepresented than dogs (Table 3) with a higher mean age of incidence (Figure 2 and Table S1). When comparing to dogs, cats show higher odds of being affected in all glands with the exception of the cranial abdominal (M3).

#### 3.2.7. Eye and Adnexa

Tumors in this group are five-fold more represented in cats than dogs without sex predilection in either species (Table 3). The mean age of incidence is not different per species or sex (Figure 2 and Table S1).

#### 3.2.8. Endocrine Glands

Thyroid gland tumors represent almost two thirds of the tumors in this group (Table 3). There is no sex predilection (Table 3) and no significant difference in mean age of incidence for species or sex (Figure 2 and Table S1).

#### 3.2.9. Connective, Subcutaneous and Other Soft Tissues

Dogs are two-fold more likely to have a tumor in this group of topographies without sex predilection (Table 3). Cats show a higher mean age of incidence than dogs, mainly due to the contribution of male cats (Figure 2 and Table S1).

#### 3.2.10. Lymph Nodes

Cats are two-fold more represented than dogs without sex predilection in both species (Table 3). Regarding mean age: cats show a higher mean age than dogs; only in dogs, females show a higher mean age than males; and females of both species grouped together, showed a higher mean age than males (Figure 2 and Table S1).

#### 3.2.11. Bones and Joints

Tumors in bones did not show species’ predilection or difference in mean age of incidence. However, male dogs show a higher proportion than females (Table 3 and Table S1, Figure 2).

#### 3.2.12. Skin

Table 3 shows that dogs have 1.75-fold higher overall odds of having skin tumors than cats both with a higher male proportion, more pronounced in cats. Cats showed 3.4 times the odds than dogs of presenting tumors in the external ear and in the skin of the face, whereas dogs are overrepresented compared to cats in eyelid, forelimbs and hind limbs. Moreover, perianal skin tumors dominantly affect male dogs (Table 3). Regarding the mean age of incidence of skin tumors (Figure 2 and Table S1), cats show a higher mean age than dogs both in females and in males. However, there are no difference between sex intra species.

### 3.3. Morphologies’ Analysis

The results presented focus on a selection of 7245 tumors records available, which were classified in a total of 245 different morphologies. The selected cases are presented on Table 4. In the overall sample, malignant mammary (MM) subtypes were the most frequent (*n* = 1186, 13.1%), followed by benign mammary (BM) subtypes (*n* = 1040, 11.5%), mast cell tumors (MCT) (*n* = 1014, 11.2%), lipomas (*n* = 452, 5.0%), squamous cell carcinoma (SCC) (*n* = 379, 4.2%), lymphomas (*n* = 302, 3.3%) and histiocytomas (*n* = 270, 3.0%).

Table 4 shows that dogs were more likely (*p* < 0.05) to present neoplasms of histiocytic origins, MCT, melanomas and melanocytomas, BM tumors, blood vessel tumors (BVT), and lipomas than cats. Cats were overrepresented (*p* < 0.05) in T-cell lymphomas, MM tumors, SCC, overall lymphomas, fibrosarcomas, B-cell lymphomas and overall mammary tumors. In dogs, although not in cats, lipomas, SCC, overall lymphomas and osseous neoplasms do not have sex predilection.

#### 3.3.1. Mammary Tumors

**Figure 3:**
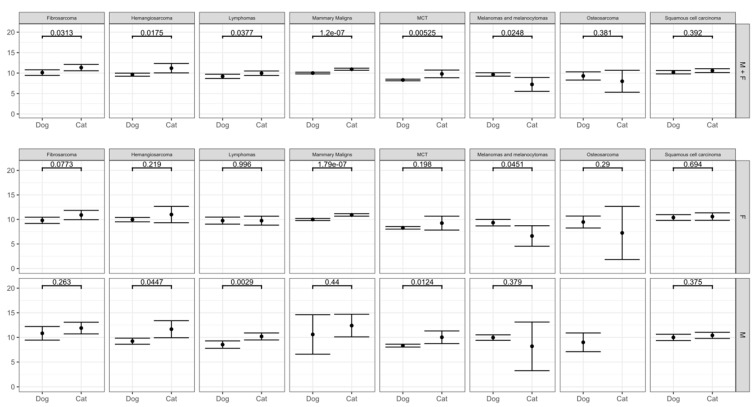
Mean age distribution of main morphologies by species and sex with the respective *p*-value.

Compared to dogs, cats are two-fold overrepresented in mammary tumors and five-fold in the malignant subtypes (Table 4). Mean age of detection is also higher for cats than for dogs (Figure 3 and Table S2). There were no differences in mean age of incidence for BM tumors by species. However, MM tumors showed an older mean age of incidence in cats. (Figure 3 and Table S2).

#### 3.3.2. Mast Cell Tumors

MCT occurs almost four-fold higher in dogs with a predominance of males in both species (Table 4). Cats present a higher mean age of incidence compared to dogs, however, without differences within sex either for dogs or for cats (Figure 3 and Table S2).

#### 3.3.3. Lipomas

Dogs were 1.8 times more likely to have lipomas, although without sex predilection (Table 4). On the other hand, the proportion of lipomas in male cats is two times higher than in females. Regarding mean age of incidence, cats showed a higher mean than dogs and there were no differences within species’ sex (Figure 3 and Table S2).

#### 3.3.4. Blood Vessel Tumors

Both hemangiomas and hemangiosarcomas are more represented in dogs than in cats. In both species, males have higher proportions (Table 4). Cats have higher mean age of hemangiosarcomas incidence than dogs and when grouped together, females showed a higher mean age than males (Figure 3 and Table S2).

#### 3.3.5. Neoplasms of Histiocytes

Dogs have ten times the odds than cats of having a tumor in this group (Table 4). Cutaneous histiocytoma is responsible for such difference, being at 1.6 higher proportion in male than female dogs. On the contrary, female dogs showed 4.9-fold higher proportion than males of presenting histiocytic sarcoma. Canine histiocytomas presented the lowest mean age of incidence all tumors of 3.8 y.o (SD 2.9) with no significant difference for sex (Table S2).

#### 3.3.6. Melanomas and Melanocytomas

Dogs have almost five times the odds of presenting melanomas than cats, with males showing two-fold higher proportion than females, which is not seen in cats (Table 4). There are only two cases of amelanotic melanomas in cats. Melanocytomas were reported 1.6-fold higher in male than in female dogs (Table 4). Different from other topography groups, cats present a lower mean age of incidence than dogs (Figure 3 and Table S2).

#### 3.3.7. Canine Perivascular Wall Tumors

This subtype represents more than 3% of all canine tumors with 1.3-fold male predilection (Table 4). The mean age of incidence is 10.1 years old (SD 2.7) without difference by sex (Table S2).

#### 3.3.8. Squamous Cell Carcinoma

Cats have five times the odds compared to dogs with a two-fold male predilection (Table 4). There are no differences in mean age of incidence by species or sex (Figure 3 and Table S2).

#### 3.3.9. Lymphomas

Cats are 4.5-fold more represented than dogs in lymphomas’ overall group and six times the odds for T-cell lymphomas subtypes. Males from both species showed a higher proportion than females more pronounced in cats with 2.7-fold (Table 4). The mean age of incidence is lower in males when compared to female dogs and overall higher in cats (Figure 3 and Table S2).

#### 3.3.10. Fibrosarcomas

Cats are almost three times more likely to have fibrosarcomas than dogs, with higher male proportion for both species, although more pronounced in cats (Table 4). Cats present a higher mean age of incidence than dogs (Figure 3 and Table S2).

#### 3.3.11. Osseous Neoplasms

There was no difference for dogs and cats even in these tumors’ frequency and for mean ages. Only in cats, there are two-fold higher proportion of males (Table 4, Figure 3 and Table S2).

### 3.4. Multivariate Analysis

**Figure 4:**
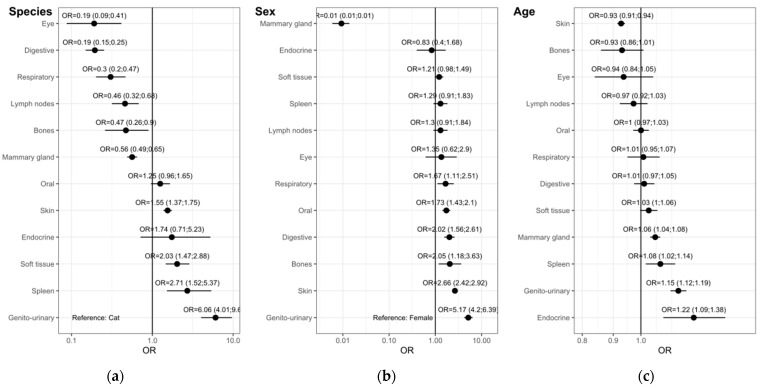
Multivariate binary logistic regression model of topography groups. Odds ratios (OR) with 95% confidence intervals (CI) adjusted by (**a**) sex and age, (**b**) species and age and (**c**) species and sex.

The multivariate analysis for the topography groups (Figure 4) shows that when adjusted for sex and age (Figure 4a), dogs still have higher odds of having tumors in the genito-urinary organs, spleen, soft tissue and skin. In the other hand, cats show higher odds for tumors in the eyes, digestive, respiratory organs, lymph nodes, bones and mammary gland. Only tumors in the oral cavity have no species predilection.

Multivariate analysis adjusted for species and age (Figure 4b), shows that females have higher odds of having tumors only in the mammary gland. Soft tissue, spleen, lymph nodes and eye tumors have no sex predilection. Males have higher odds for tumors in the respiratory, oral, digestive, bones, skin and genito-urinary organs.

Multivariate analysis adjusted for species and sex (Figure 4c), shows the influence of age of having tumors in the topography groups. Tumors in the soft tissue, mammary gland, spleen and genito-urinary tumors show higher odds with the increase in age. Tumors in the bone, eye, lymph nodes, oral cavity, respiratory and digestive organs seem not to be influenced by aging. In our data, the odds of having a skin tumor decrease with age.

**Figure 5:**
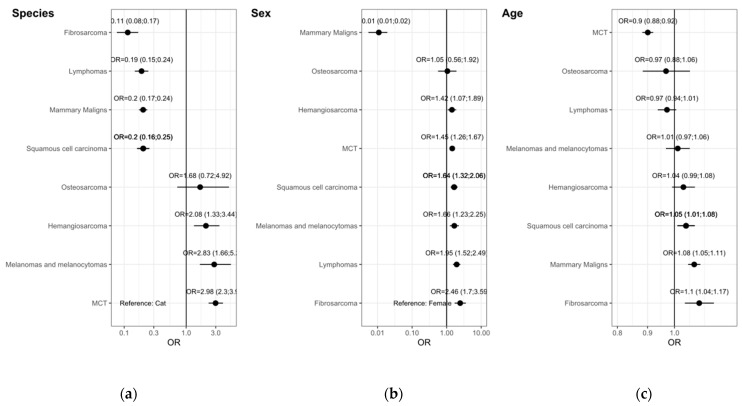
Multivariate binary logistic regression model for main morphologies. Odds ratios (OR) with 95% confidence intervals (CI) adjusted by (**a**) sex and age, (**b**) species and age and (**c**) species and sex.

The multivariate analysis for the main morphologies (Figure 5) shows that when adjusted for sex and age (Figure 5a), dogs have higher odds of having MCT, melanomas and melanocytomas and hemangiosarcomas while cats have higher odds for fibrosarcomas, lymphomas, MM and SCC. Multivariate analysis adjusted for species and age (Figure 5b), shows that males have higher odds for all morphologies listed, with the exception for mammary tumors and osteosarcomas, this showing no sex predilection. Figure 4c, multivariate analysis adjusted for species and sex, shows that only SCC, MM tumors and fibrosarcomas show higher odds with the increase in age. In our data, the odds of having an MCT decreases with age.

## 4. Discussion

To the authors’ knowledge, the comparison undertaken in this work, concerning the broad comparison of tumors incidence among dogs and cats, is absent in the literature. Performing analysis over a dataset including all the tumors reported from various laboratories operating at the country level, without exclusions of tumor types, allows one to perform comparative oncology on the frequencies between both species. This approach is useful as it may reveal species specificities due to differences regarding different profiles of topography, morphology, and age of incidence in tumor occurrence, which can suggest the contribution of genetic or behavioral determinants besides the environmental ones typically mentioned in reports. Since cats and dogs present in the database share roughly the same domestic environment and come, haphazard, from the same territory, environmental exposures are probably not the only explanatory factor contributing to the differences observed in our dataset in tumor type profiles, opening the door for the involvement of genetic or behavioral determinants. The analysis of cancer distribution in dogs and cats revealed a predominance of tumors in dogs, in females from both species, in the skin and mammary gland, which is in accordance with previous studies from other countries [21,22,23].

### 4.1. Age of Incidence

Focusing on the age of presentation of tumors it is clear that from birth to seven years old, male dogs present earlier incidence, and from birth to eight years old, cat females show later incidence than others. From those ages onwards, the age of incidence in sex within species becomes indistinguishable, remaining different among species, with cats later than dogs. This could be related with longer life expectancy in cats than in dogs. In our sample cats tend to present tumors one year later than dogs, with this happening either in females or in males. The topographies most contributing to the differences were the oral cavity, mammary gland, soft tissue, lymph nodes and skin, where cats always show the incidence at a latter age than dogs. Only in cats is a sex over representation seen for certain tumors: cat females are older than males in oral cavity and lymph nodes, and the reverse is true for genito-urinary and soft tissue. Although the literature reports some age associated cancer incidence in dogs and cats [24], the lack of comparative studies between the two species prevents further discussion on present data.

### 4.2. Profiling the Risk of Tumor Presentation from Both Species

Regarding species representation by topography, a distinct pattern between the two species was observed. Dogs are overrepresented in genito-urinary, spleen, soft tissue, skin and oral and pharyngeal cavity, whereas cats are overrepresented in mammary gland, lymph nodes, respiratory, digestive and eye and adnexa. Moving to the main morphologies, the species profile is also interesting: dogs are overrepresented in neoplasms of histiocytes, blood vessel tumors (hemangiomas and hemangiosarcomas), mast cell tumors, melanomas and melanocytomas, benign mammary tumors and lipomas. Cats, on their side, are overrepresented in lymphomas in general, either T or B-cell lymphoma, in malignant mammary tumor and mammary tumor in general, fibrosarcoma and squamous cell carcinoma.

Within the species, the profile observed was not very different from that reported in other studies. Dogs presented more tumors on skin (almost 50% of all dog tumors) similar to other studies [21,23], followed by mammary gland (23.1%) and genito-urinary (7,7%). Skin tumors in dogs are mainly reported in males in unspecific sites (Skin, NOS, 20.5%), followed by skin trunk (8.2%) and skin rear limb (5.0%). Genito-urinary tumors are most represented by testis tumors (59.4%) which is accordance with other studies [21,25]. Gum tumors are also highly represented in this study (3.2%) mainly in male dogs. In contrast, felines have more tumors in tongue, although a low number of cases is involved. For cats, the most affected topography was the mammary gland (38.1%) followed by skin (36.1%), with tumors in the skin of the face, skin of the trunk and skin of external ears the preferred localization. Tumors on the digestive organs (8.2%) of cats occupies the third place in topography analysis, taking special attention for tumors in the intestinal tract (6.4%) similar to the previous study from Swiss Cancer registry (7.5%) [26] specially in male cats.

### 4.3. Detailed Assessment of Results

Mammary gland tumors were divided into benign and malignant and, also, separated per gland affected. Malignant mammary tumors are most represented in cats with a higher mean age of incidence. The most affected glands were caudal abdominal and inguinal in dogs, however, in cats it was mainly the cranial abdominal gland, which is similar to other studies [27,28]. There are no similar studies reported in the literature concerning comparative incidences and anatomical location of mammary cancer on the two species, however it is well known that there is a high prevalence of malignant mammary tumors in cats [29], which is corroborated in this study. The neutering status of the animal can influence mammary gland tumors to occur, and since our data was not complete regarding this information, the factor was not included in the analysis. Noticeable from Table 4 is that, irrespective of the prevalence of neutering in dogs or cats, malignancy is very different in proportion. This evidence deserves attention in future epidemiological studies.

Mast cell tumors are more represented in young male dogs, in accordance with other studies [30,31,32]. As reported in other studies, blood vessel tumors are more likely to appear in dogs, more frequently in males and appear later in females [21,33]. Histiocytomas are, in particular for male dogs, associated with the earliest mean age of incidence [28]. Genital tumors are practically absent in cats with the exception of tumors in the feline uterus. The absence of testicular tumors in cats may reflect the decision of castrate cats to avoid the urine-marking behavior [34].

Lymphomas are more frequent in cats, in males without aging influence. It was found in other studies that risk of lymphoma increases with age until six years and decrease thereafter [26,32,35,36,37]. Lymphomas in cats are indeed very common, particularly intestinal lymphoma, probably not associated with Feline leukemia virus (FeLV) since it has been reported that FeLV is typically associated with mediastinal (thymic) lymphomas [38,39].

In our data, osteosarcomas show sex predilection similar to data from other studies [40,41]. However, our data did not present a difference between species and the aging seems not to increase the odds for this tumor. This can be seen as odd in the light of other studies referring higher frequency in dogs [21,40]. The recollection of additional data in the coming years in our system will allow us to clarify whether this finding stands.

Although fibrosarcomas occur in all species of domestic animals, they are most commonly seen in adult and elderly cats and dogs. The estimated incidence of fibrosarcoma in dogs in the United States is approximately 3% [42], higher than that shown in this study (1.53%). In cats, fibrosarcoma is the most common tumor and its incidence has increased over the past two decades, likely due to its association with vaccination [27]. There is no known gender predisposition reported in cats [27], however, males are overrepresented in our study.

SCC is the second most common neoplasia in cats similar to other studies [26,43]. Males and aging are factors positively with SCC in our study, in accordance with previously reported [43].

The analysis of our results reveals significant differences in the global mean incidence age: cats show higher incidence mean age than dogs in general. Specifically, cats showed higher mean age of incidence for oral, mammary gland, soft tissue, lymph nodes and skin, whereas for all other topographies, age is not different. Concerning morphology, except for osteosarcoma, melanoma and SCC tumors, in all other groups, cats showed a higher mean age. Conversely, dogs showed a higher mean age in melanomas and melanocytomas.

The results entering the database did not suffer from of any type of exclusion before entering the analysis, the relative proportion of veterinary sample submissions to the laboratories was respected, hence age and species frequencies shall represent the tumor occurrence from the ground fairly well. Companion animals, due to their close coexistence with their owners, share the same environmental, social, economic and cultural characteristics that may influence the occurrence of neoplasms in particular regions or countries. Rural and urban environments also allow contact with different environmental factors that can influence the type and frequency of cancer [6,35,44,45,46,47]. Since no exclusion criteria were performed on the data from both species come, haphazard, from the same territory. Probably, environmental exposures are not the only explanatory factor contributing to the differences observed in our dataset in tumor type profiles, opening the door for the involvement of genetic or behavioral determinants. Since cats come from a distinct taxonomic family, *Felidae*, and dogs, *Canidae*, two distinct suborders of the Carnivora, therefore, showing distinct genetic signatures [48,49], a genetic contribution for the differences observed cannot be overruled.

### 4.4. Limitations and External Validity

Our database harbors the complete set of results from two years of work from our VLP partners, however their results are not the full set of results produced in Portugal. We estimate that our data covers three quarters of results produced annually, covering all the Portuguese territory since the VLP work clients at country level, not regional. Although full representativeness of the sample cannot be claimed, is fair to assume that VLP clients are not biased by region. The frequency and distribution of the source population of dogs and cats in Portugal and their demographic structure is not available at present, which makes it difficult to dig deeper into the issue of representativeness. This limitation also resulted in the impossibility of calculating incidence proportions. Hence, the proportions calculated allows for the comparison of the relative presence of a tumor of a certain type among all the tumors of the species not accounting for the population frequencies. The full external validation of our results is therefore excluded.

It is worth noting that this is an epidemiological study focused on the effect, that is, the presence of the tumor in relation to type, sex and age. The reasons why certain proportions are observed are debatable, and variables that help explain the evidence provided are often missing. This limits the conclusions and provides an opportunity to improve the registry for future studies.

The reasons for a lower frequency of tumors in cats than in dogs could be due to factors contributing to lower reporting, like, for example, the owners of cats attending veterinary clinics less often, more stray cats than dogs, or the existence of less cats than dogs in the population. Dog and cat population figures for Portugal are, respectively, 2.0 and 1.5 million [1], which allows us to disregard the population bias possibility.

Another limitation could be related to the full comparability of results produced by several pathologists from the different laboratories. This limitation is generally accepted as affecting comparisons between laboratories, in the absence of accepted general standards. However, a standardization and quality assessment step of the crude results was introduced in our system before their integration into the database; this step was performed by one author, and always by that same author, who was in close contact with the pathologists to clarify possible inconsistences present at the data level when reported.

## 5. Conclusions

Our study shows remarkable differences in tumor presentation between dogs and cats adjusted by age and sex, regarding tumor topography, and morphology. Based on this evidence it was suggested that there were different tumor risk profiles for dogs and cats. These differences allow us to raise the hypothesis of possible genetic or behavioral contributions at a suborder level, beyond environmental factors, to explain tumor occurrence differences between those species.

Within the species, the profile observed regarding topographies, morphologies and incidence age in tumors was not very different from findings already reported in other studies. However, the differences between species are not so often investigated and our study is a pioneering contribution within this domain.

In future research relating genetic, epigenetic and omics expression comparative oncology involving human and other species, cats could be considered besides dogs, since they seem to be more prone to lymphomas in general, either T or B-cell lymphoma, in malignant mammary tumor and mammary tumor in general, fibrosarcoma and squamous cell carcinoma than dogs.

Regarding the conclusions from our study, please bear in mind the limitations discussed above, and recognize this study as an epidemiological contribution to the comparative oncology field.

This study marks the beginning of a continuous animal cancer registration system in Portugal, with which the authors expect to positively contribute to the research on veterinary and comparative oncology.

## Figures and Tables

**Table 1:** Description of topography groups and respective belonging topography codes.

Topography Groups	Short Name	Correspondence with ICD-O-3.2 Topography Codes
Oral and Pharyngeal Cavity	Oral	C00. to C14.
Peritoneum and Digestive Organs	Digestive	C15. to C26.; C48.
Respiratory System, Intrathoracic Organs and Midlle ear	Respiratory	C30C39
Hematopoietic System and Endothelial Reticulum	Spleen	C42.
Genito-Urinary Organs	Genito-urinary	C51. to C68.
Mammary gland	Mammary	C50.
Eye and Adnexa	Eye	C69.
Nervous system	Nervous	C47.; C70 to C72.
Endocrine Glands	Endocrine	C73. to C75.
Connective, subcutaneous and other soft tissues	Soft tissue	C49.
Lymph nodes	Lymph nodes	C77.
Bones and Joints	Bones	C40. to C41.
Unknown primary site	UKN	C80.
Skin	Skin	C44.

**Table 2:** Analysis of tumors by species, sex (total number, n, and proportion, %) and age (mean age and standard deviation).

Tumors in (*n*, %)	Dogs (7245, 81.1)	Cats (1689, 18.9)	Total (8934)
Number of females	4245 (58.6 ^a;c^)	1138 (67.4 ^b;c^)	5383 (60.2 ^c^)
Number of males	3000 (41.4 ^a^)	551 (32.6 ^b^)	3551 (39.8)
Mean age of incidence (SD) ^Min–max^			Difference (CI95%)
All animals	9.3 ^a^ (3.1) ^0.25–20^	10.5 ^b^ (3.4)^0.5–20^	1.2 (1.07–1.40)
Females	9.3 ^a^ (3.0) ^0.25–18^	10.6 ^b^ (3.3) ^0.5–19^	1.3 (1.09–1.50)
Males	9.2 ^a^ (3.4) ^0.25–20^	10.4 ^b^ (3.6) ^0.5–20^	1.2 (0.89–1.5)

Proportion comparison between sex.: Z-test difference for proportion, *p* < 0.001. Letter a and b signals significant differences among sex between species (difference in rows). Cells marked with letter c signal differences between sex within species (in columns). T-test difference in mean ages, *p* < 0.001.

**Table 3:** Univariate analysis by topography groups (bold) and topography item (underneath) of dogs and cats with number of cases (*n*), proportion from total within each species (%), sex proportion ratio (PR, female/male) with the respective CI 95%, crude Odds Ratio (OR) of dogs/cats and respective *p*-value.

Topography Groups	Tumors in Dogs (*n* = 7355)			Tumors in Cats (*n* = 1724)				
Topography Item	*n*	%	Sex PR ^(f/m)^ (CI 95%)	*n*	%	Sex PR ^(f/m)^ (CI 95%)	OR ^dog/cat^	*p*-Value ^!^
**Oral and Pharyngeal Cavity**	**417**	**5.8** ^$^	0.6 (0.49–0.71) *	**73**	**4.3** ^$^	0.6 (0.39–0.97) *	**1.32**	**0.033**
Gum	230	55.2	0.6 (0.45–0.75) *	24	32.9	0.5 (0.22–1.07)	2.30	<0.001
Lip	76	18.2	0.5 (0.31–0.77) *	14	19.2	0.5 (0.17–1.37)	1.28	0.484
Tongue	18	4.3	1.1 (0.43-2.86)	12	16.4	1.0 (0.29–3.20)	0.37	0.010
Mouth, NOS	84	20.1	0.6 (0.38–0.89) *	19	26.0	0.7 (0.27–1.64)	1.00	0.994
**Peritoneum and Digestive Organs**	**131**	**1.8** ^$^	**0.7 (0.51–1.01)**	**138**	**8.2** ^$^	0.4 (0.25–0.48) *	**0.21**	**<0.001**
Stomach	20	15.3	0.6 (0.24–1.39)	11	8.0	0.1 (0.02–0.50) *	0.42	0.034
Intestinal tract	63	48.1	0.5 (0.28–0.77) *	110	79.7	0.3 (0.22–0.47) *	0.13	<0.001
Liver	40	30.5	1.6 (0.84–3.24)	13	9.4	1.1 (0.34–3.52)	0.23	0.292
**Respiratory System, Intrathoracic Organs**	**62**	**0.9** ^$^	0.5 (0.33–0.90) *	**45**	**2.7** ^$^	**0.6 (0.34–1.08)**	**0.31**	**<0.001**
Nasal cavity	21	33.9	0.5 (0.22–1.26)	19	42.2	0.5 (0.22–1.31)	0.26	<0.001
**Hematopoietic System and Endothelial Reticulum–Spleen**	**127**	**1.8** ^$^	**0.7 (0.51–1.01)**	**12**	**0.7** ^$^	**1.4 (0.39–5.33)**	**2.57**	**<0.001**
**Genito-Urinary Organs**	**555**	**7.7** ^$^	0.2 (0.15–0.23) *	**24**	**1.4** ^$^	**1.4 (0.58–3.63)**	**5.58**	**<0.001**
Bladder	15	2.7	1.9 (0.62–6.10)	5	20.8	all cases in males	0.75	0.775
Kidney	11	2.0	0.6 (0.18–1.93)	3	12.5	all case in females	0.86	0.914
Urethra	7	1.3	4.2 (0.51–35.19)	0	0.0	-		
*Male Genital organs*	430	77.5	-	1	4.2	-	106.7	<0.001
Testis	330	76.7	-	1	100	-	80.9	<0.001
Scrotum	60	14.0	-	0	0.0	-		
Penis	35	8.1	-	0	0.0	-		
*Female Genital organs*	93	16.8	-	15	62.5	-	1.46	0.216
Ovary	31	33.3	-	2	13.3	-	3.64	0.094
Uterus	17	18.3	-	12	80.0	-	0.33	0.004
Vagina	27	29.0	-	0	0.0	-		
Vulva	16	17.2	-	0	0.0	-		
**Mammary Gland**	**1670**	**23.1** ^$^	61.4 (39.15–96.2) *	**643**	**38.1** ^$^	38.4 (19.24–76.47) *	**0.50**	**<0.001**
Cranial thoracic	23	1.4	15.5 (2.10–115.2) *	19	3.0	-	0.28	<0.001
Caudal thoracic	69	4.1	48.0 (6.67–345.7) *	39	6.1	-	0.41	<0.001
Cranial abdominal	92	5.5	31.8 (7.84–128.9) *	70	10.9	-	3.43	<0.001
Caudal abdominal	143	8.6	100.3 (14.04–716.6) *	43	6.7	11.4 (2.77–46.59) *	0.69	0.023
Inguinal	142	8.5	99.6 (13.94–711.6) *	0	0.0	-		
Nipple	2	0.1	0.7 (0.04–11.29)	0	0.0	-		
Mammary gland. NOS	1069	64.0	67.9 (37.58–122.8) *	407	63.3	32.3 (14.52–71.86) *	0.55	<0.001
Overlapping lesion of mammary gland ^a^	130	7.8	91.1 (12.75–651.5) *	59	9.2	-	0.51	<0.001
**Eye and Adnexa**	**18**	**0.2** ^$^	**0.6 (0.22–1.43)**	**20**	**1.2** ^$^	**0.6 (0.25–1.42)**	**0.20**	**<0.001**
**Nervous system**-Spinal cord	1	0.0 ^$^	-	0	0.0 ^$^	-		
**Endocrine Glands**	**32**	**0.4** ^$^	**1.8 (0.84–3.90)**	**7**	**0.4** ^$^	**0.4 (0.08–1.61)**	**1.07**	**0.969**
Adrenal gland	8	25.0	0.7 (0.18–2.82)	2	28.6	0.5 (0.03–7.71)	0.94	0.748
Thyroid gland	21	65.6	2.3 (0.83–6.16)	5	71.4	0.3 (0.05–1.92)	0.98	0.827
**Connective, subcutaneous and other soft tissues**	**417**	**5.8** ^$^	**0.9 (0.76–1.10)**	**43**	**2.5** ^$^	**0.7 (0.37–1.22)**	**2.21**	**<0.001**
**Lymph Nodes**	**94**	**1.3** ^$^	**0.7 (0.49–1.10)**	**45**	**2.7** ^$^	**1.0 (0.52–1.78)**	**0.46**	**<0.001**
**Bones and Joints**	**43**	**0.6** ^$^	0.3 (0.25–0.85) *	**16**	**0.9** ^$^	**0.8 (0.29–2.20)**	**0.65**	**0.164**
**Unknown primary site**	**71**	**0.9** ^$^	0.6 (0.35–0.90) *	**13**	**0.8** ^$^	**0.4 (0.14–1.23)**	**1.30**	**0.463**
**Skin**	**3604**	**49.7** ^$^	0.7 (0.65–0.71) *	**609**	**36.1** ^$^	0.4 (0.33–0.43) *	**1.75**	**<0.001**
External ear	80	2.2	0.5 (0.34–0.81)	64	10.5	0.5 (0.32–0.83) *	0.29	<0.001
Eyelid	201	5.6	0.6 (0.47–0.82) *	26	4.3	0.6 (0.26–1.21)	1.87	<0.001
Skin of face	288	8.0	0.6 (0.50–0.78) *	128	21.0	2.0 (1.11–3.65) *	0.49	<0.001
Skin of forelimb	178	4.9	0.6 (0.46–0.83) *	27	4.4	0.5 (0.25–1.10)	1.62	0.024
Skin of hindlimb	364	10.1	0.8 (0.65–0.96) *	31	5.1	0.5 (0.26–1.04)	2.93	<0.001
Skin lip	8	0.2	0.2 (0.05–1.17)	2	0.3	0.5 (0.03–7.71)	0.94	0.748
Skin of occiput, nape and neck	92	2.6	0.6 (0.41–0.93) *	16	2.6	0.5 (0.18–1.28)	1.41	0.248
Skin trunk	593	16.5	0.6 (0.56–0.74) *	73	12.0	0.3 (0.20–0.50) *	1.93	<0.001
Skin, NOS	1485	41.2	0.8 (0.70–0.84) *	232	38.1	0.3 (0.26–0.42) *	1.63	<0.001
Skin of tail	29	0.8	0.9 (0.42–1.80)	6	1.0	0.2 (0.04–1.32)	1.00	0.842
Perianal skin	285	7.9	0.2 (0.15–0.26)	3	0.5	0.2 (0.02–2.66)	23.9	<0.001

Legend of symbols: ^$^ Proportions from the total of the respective column; *: Significant CI95%; ^!^ *p*-value: underline when is lower than 0.05. NOS: no otherwise specified. ^a^ when more than two mammary glands were affected. The bold is to highlight the topography group (explained in the title).

**Table 4:** Univariate analyzes of the main morphologies of dogs and cats with number of cases (*n*), proportion from total within each species (%), sex proportion ratio (PR, female/male) with the respective CI 95%, crude Odds ratio (OR) of dogs/cats and the respective *p*-value.

Morphology	Tumors in Dogs			Tumors in Cats				
	(*n* = 7334)			(*n* = 1719)				
	*n*	%	Sex PR ^(f/m)^ (CI 95%)	*n*	%	Sex PR ^(f/m)^ (CI 95%)	OR ^dog/cat^	*p*-Value ^!^
**Mammary tumors**	1587	21.9 ^$^	92.8 (52.69–163.5) *	635	37.6 ^$^	50.8 (22.86–112.7) *	0.47	<0.001
Benigns	967	60.9	136.0 (56.57–327.2) *	73	11.5	0	3.42	<0.001
Maligns	624	39.1	61.9 (29.44–130.2) *	562	88.5	44.9 (20.20–99.66) *	0.19	<0.001
**Mast cell tumors**	949	13.1 ^$^	0.8 (0.71–0.90) *	65	3.8 ^$^	0.4 (0.23–0.61) *	3.78	<0.001
**Lipoma**	399	5.5 ^$^	1.0 (0.81–1.19)	53	3.1 ^$^	0.5 (0.31–0.89) *	1.81	<0.001
**Blood vessel tumors**	387	5.3 ^$^	0.7 (0.61–0.90) *	30	1.8 ^$^	0.6 (0.27–1.13)	3.14	<0.001
Hemangiomas	189	48.8	0.7 (0.56–0.99) *	7	23.3	0.2 (0.04–1.00) *	6.47	<0.001
Hemangiosarcoma	198	51.2	0.7 (0.55–0.96) *	23	76.7	0.8 (0.33–1.73)	2.05	0.006
**Neoplasms of histiocytes**	296	4.1 ^$^	0.6 (0.45–0.70) *	7	0.4 ^$^	2.9 (0.35–24.07)	10.29	<0.001
Cutaneous histiocytoma	270	91.2	0.5 (0.37–0.61) *	0	0.0	-		
Histiocytic sarcoma	26	8.8	4.9 (1.48–16.58) *	7	100	2.9 (0.35–24.07)	0.87	0.917
**Melanomas and melanocytomas**	245	3.3 ^$^	0.6 (0.47–0.78) *	20	1.1 ^$^	1.0 (0.37–2.57)	2.94	<0.001
Amelanotic melanoma	49	20.0	0.9 (0.50–1.52)	2	10.0	0.5 (0.03–7.73)	5.77	0.010
Melanoma, NOS	100	40.8	0.5 (0.34–0.76) *	11	55.0	1.1 (0.29–4.35)	2.15	0.020
Melanocytoma	94	38.4	0.6 (0.42–0.95) *	7	35.0	1.0 (0.18–5.24)	3.18	0.003
**Canine perivascular cell wall tumors**	237	3.3 ^$^	0.8 (0.59–0.97) *	0	0.0	-	-	-
**Squamous cell carcinoma**	182	2.5 ^$^	0.8 (0.61–1.09)	195	11.5 ^$^	0.5 (0.38–0.64) *	0.20	<0.001
**Lymphomas**	151	2.1 ^$^	0.7 (0.54–1.02)	151	8.9 ^$^	0.3 (0.25–0.46) *	0.22	<0.001
Lymphoma, non-Hodgkin, NOS	100	66.2	0.7 (0.45–0.98) *	116	76.8	0.4 (0.26–0.52) *	0.19	<0.001
Cutaneous lymphomas	24	15.9	1.2 (0.66–2.02)	4	2.6	0.2 (0.02–1.55)	1.41	0.699
B-cell lymphomas	11	7.3	0.6 (0.18–1.93)	7	4.6	1.2 (0.24–6.22)	0.37	0.064
T-cell lymphomas	15	9.9	1.1 (0.38–2.98)	24	15.9	0.2 (0.07–0.43) *	0.14	<0.001
**Fibrosarcoma**	50	1.53 ^$^	0.8 (0.59–0.97) *	95	5.6 ^$^	0.3 (0.19–0.44) *	0.12	<0.001
**Osseous neoplasms**	60	0.8 ^$^	0.7 (0.49–1.02)	9	0.5 ^$^	0.5 (0.34–0.69) *	1.57	0.267
Osteoma	5	8.3	2.8 (0.32–25.3)	0	0	-		
Osteosarcomas	55	91.7	0.8 (0.44–1.31)	9	100	0.5 (0.12–1.93)	1.44	0.396
**Other morphologies not considered in the analysis**	2474	33.7 ^$^		455	26.5			

Legend of symbols: ^$^ Proportions were calculated from the total of the respective column. * Significant CI95%; ^!^ *p*-value: underlined when is lower than 0.05. NOS: no otherwise specified.

## Data Availability

The data presented in this study are available on request from the corresponding author. The data are not publicly available due to ethics.

## Supplementary Materials

The following are available online at https://www.mdpi.com/article/10.3390/vetsci9040167/s1, Figure S1: Age distribution by species, Table S1: Age analysis of topography groups by specie and sex. Mean (SD), Table S2: Age analysis of the selected morphologies by specie and sex. Mean (SD).
