# Supplementary material for: Cross Species Analysis and Comparison of Tumors in Dogs and Cats, by Age, Sex, Topography and Main Morphologies. Data from Vet-OncoNet
Source: Vet Sci. 2022 Mar 31;9(4):167. doi: 10.3390/vetsci9040167 (PMC9025902; doi:10.3390/vetsci9040167)
Supplement: Supplementary file 1 [file vetsci-09-00167-s001.zip › vetsci-1613324-supplementary.pdf]

Supplementary materials

Cross species analysis of tumors in dogs and cats, by age, sex, topography and main morphologies. Data from Vet-OncoNet.

Katia Pinello[1,2,3*], Isabel Pires[4,5], Ana Filipa Castro[1], Paulo Tiago Carvalho[1], Andreia Santos[6,7], Augusto de Matos[6,7], Felisbina Queiroga[4,5,7], Irina Amorim[8], Ana Canadas-Sousa[8], Patrícia Dias Pereira[8], José Catarino[9,10], Pedro Faísca[9,10,11], Sandra Branco[12], Cristiana Lopes[13], Filipa Marcos[13], Maria C. Peleteiro[13], Hugo Pissarra[13], Pedro Ruivo[13], Rui Magalhães[1], Milton Severo[2,3] and João Niza-Ribeiro[1,2,3]

[1] Departamento de Estudo de Populações, Vet-OncoNet, ICBAS, Instituto de Ciências Biomédicas Abel Salazar, Universidade do Porto, Porto, Portugal.
[2] EPIUnit - Instituto de Saúde Pública, Universidade do Porto, Porto, Portugal
[3] Laboratório para a Investigação Integrativa e Translacional em Saúde Populacional (ITR), Porto, Portugal.
[4] Departamento de Ciências Veterinárias, Universidade Trás-os-Montes e Alto Douro (UTAD), Vila Real, Portugal.
[5] CECAV - Centro de Ciência Animal e Veterinária , Universidade de Trás-os-Montes e Alto Douro, Vila Real, Portugal
[6] Departamento de Clínicas Veterinárias, ICBAS, Instituto de Ciências Biomédicas de Abel Salazar, Universidade do Porto, Porto, Portugal.
[7] CECA-ICETA - Centro de Estudos de Ciência Animal, Instituto de Ciências, Tecnologias e Agroambiente, Universidade do Porto, Porto, Portugal.
[8] Departamento de Patologia e Imunologia Molecular, ICBAS, Instituto de Ciências Biomédicas Abel Salazar, Universidade do Porto, Porto, Portugal.
[9] DNAtech, Veterinary Laboratory, Lisboa, Portugal.
[10] Faculdade de Medicina Veterinária, Universidade Lusófona de Humanidades e Tecnologia, Lisboa, Portugal
[11] CBIOS- Research Center for Biosciences & Health Technologies, Universidade Lusófona de Humanidades e Tecnologia, Lisboa, Portugal.
[12] Instituto Mediterrâneo para a Agricultura, Ambiente e Desenvolvimento, MED, Universidade de Évora, Évora, Portugal
[13] VetPat, Veterinary Pathology Laboratory, Lisboa, Portugal.
* Correspondence: kcpinello@icbas.up.pt

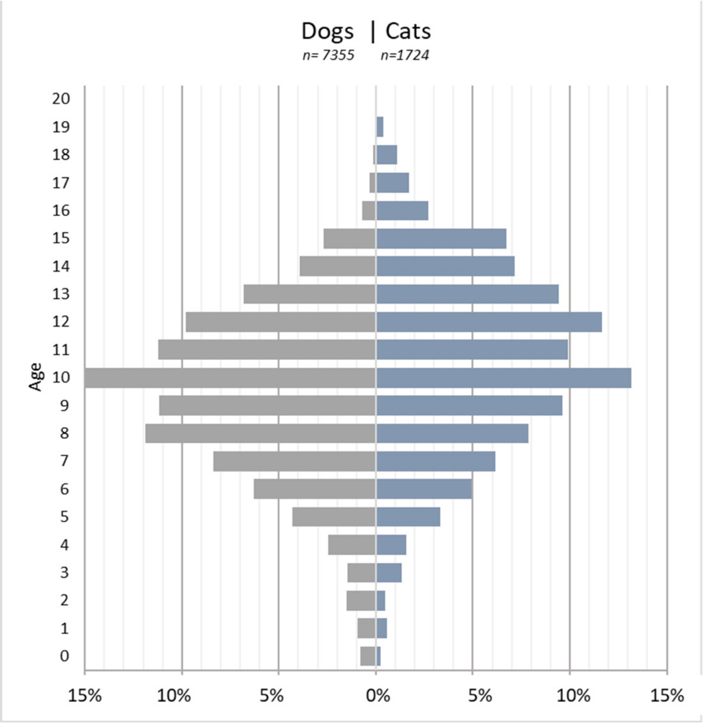

Figure S1. Age distribution by species**.**

Table S1. Age analysis of topography groups by specie and sex. Mean (SD).

	**Total**			**Dog**			**Cat**		
	**total**	**female**	**male**	**total**	**female**	**male**	**total**	**female**	**male**
1_Oral Cavity	9.5 (3.6)	9.6 (3.7)	9.3 (3.4)	9.2 (3.4)*	9.1 (3.5) f	9.2 (3.3)	11.4 (3.9)	12.3 (3.7)	10.2 (3.9)#
2_Digestive	10.0 (3.2)	10.0 (3.0)	10.0 (3.3)	9.7 (3.1)	10.0 (2.9)	9.4 (3.2)	10.3 (3.2)	10.1 (3.2)	10.5 (3.3)
3_Respiratory	9.9 (4.0)	9.6 (4.0)	10.1 (4.0)	9.5 (3.4)	8.9 (3.0)	9.9 (3.7)	10.4 (4.7)	10.3 (4.9)	10.4 (4.6)
4_Spleen	10.1 (2.6)	10.0 (2.5)	10.2 (2.6)	10.2 (2.4)	10.0 (2.4)	10.4 (2.4)	9.3 (4.2)	10.0 (3.6)	6.0 (7.1)
5_Genito-Urinary Organs	10.6 (2.8)	10.3 (3.2)	10.6 (2.6)	10.6 (2.8)	10.4 (3.3)	10.6 (2.6)	10.7 (2.9)	9.6 (2.7)	13.0 (1.7)#
6_Mammary Gland	10.0 (2.8)	9.9 (2.8)	9.6 (3.5)	9.7 (2.6) *	9.7 (2.6)	8.9 (3.4)	10.8 (3.0)	10.8 (3.0)	11.1 (3.3)
7_Eye	9.2 (3.7)	8.2 (3.3)	10.4 (3.9)	9.6 (3.9)	7.7 (2.4)	11.3 (4.5)	9.6 (3.6)	8.6 (4.0)	9.5 (3.2)
9_Endocrine	11.3 (2.5)	11.6 (2.6)	10.9 (2.3)	11.4 (2.4)	11.6 (2.6)	10.6 (1.5)	11.2 (3.3)	10.0 (0.0)	11.5 (3.7)
10_Soft tissue	9.6 (3.0)	9.6 (2.8)	9.6 (3.0)	9.4 (2.8) *	9.5 (2.8)	9.2 (2.7)	11.6 (3.8)	10.0 (3.3)	13.5 (3.7) #
11_Lymph Nodes	9.4 (3.3)	10.2 (3.0)	8.3 (3.2) &	8.7 (3.2) *	9.6 (3.0)	7.8 (3.3)#	10.7 (3.0)	11.2 (3.0)	10.1 (2.7)
12_Bones	8.8 (3.6)	8.4 (4.1)	9.1 (3.2)	8.8 (3.5)	8.3 (4.0)	8.9 (3.4)	9.1 (3.7)	8.7 (4.6)	9.8 (2.5)
14_Skin	9.1 (3.4)	9.0 (3.4)	9.0 (3.6)	8.9 (3.4) *	8.9 (3.2)	8.8 (3.6)	10.3 (3.6)	10.3 (3.5)	10.3 (3.6)
**Total**	**9.5 (3.2)**	**9.5 (3.0)**	**9.4 (3.5)**	**9.3 (3.2)**	**9.3 (3.0)**	**9.2 (3.4)**	**10.6 (3.4)**	**10.6 (3.3)**	**10.4 (3.6)**

* difference on the mean age between species (total dog versus total cat), p<0.05. # difference on mean age between female versus male intraspecie, p<0.05. & difference on mean age between sex (Total female versus Total cat), p<0.05.

Table S2. Age analysis of the selected morphologies by specie and sex. Mean (SD).

	**Total**			**Dog**			**Cat**		
	**total**	**female**	**male**	**total**	**female**	**male**	**total**	**female**	**male**
Mammary tumors	10.0 (2.8)	10.0 (2.8)	10.6 (3.2)	9.7 (2.6) *	9.7 (2.6)	9.7 (3.4)	10.8 (3.0)	10.8 (3.0)	12.4 (2.2)
Benigns	9.5 (2.6)	9.5 (2.6)	8.5 (1.7)	9.5 (2.6)	9.5 (2.5)	8.5 (1.7)	9.5 (2.7)	9.5 (2.7)	-
Maligns	10.4 (2.8)	10.4 (2.8)	11.5 (3.4)	9.9 (2.7) *	9.9 (2.7)	10.6 (4.3)	10.9 (3.0)	10.9 (3.0)	12.4 (2.2)
Mast cell tumors	8.4 (3.1)	8.3 (2.9)	8.5 (3.2)	8.3 (3.0)*	8.2 (2.9)	8.3 (3.1)	9.7 (3.7)	9.2 (3.6)	10.0 (3.7)
Lipoma	9.5 (2.5)	9.5 (2.4)	9.5 (2.7)	9.4 (2.4)	9.4 (2.3)	9.4 (2.7)	10.3 (3.0)	10.4 (3.0)	10.2 (3.0)
Hemangiomas	9.2 (3.1)	8.6 (2.8)	9.8 (3.1)	9.0 (2.9)	8.5 (2.8)	9.5 (2.9)	15.0 (1.3)	15.0 (0.0)	15.0 (1.4)
Hemangiosarcoma	9.7 (2.7)	10.1(2.3)	9.4 (3.0)&	9.6 (2.7) *	9.9 (2.2)	9.2 (3.0)	11.2 (2.6)	11.0 (2.8)	11.7 (2.2)
Cutaneous histiocytoma	-	-	-	3.7 (2.9)	3.8 (2.9)	3.7 (2.9)	-	-	-
Histiocytic sarcoma	8.2 (3.0)	8.2 (3.1)	8.3 (2.9)	7.9 (2.8)	7.6 (2.9)	9.7 (0.6)	9.1 (3.5)	10.0 (3.0)	4.0 (0)
Melanomas and melanocytomas	9.8 (2.9)	9.5 (3.0)	10.0 (2.8)	9.9 (2.8)*	9.8 (2.9)	10.1 (2.7)	7.2 (3.2)	6.5 (2.9)	8.3 (3.5)
Canine perivascular cell wall tumors	-	-	-	10.1 (2.7)	10.1 (2.8)	10.1 (2.6)	-	-	-
Squamous cell carcinoma	10.3 (3.1)	10.4 (3.3)	10.3 (3.0)	10.2 (2.9)	10.4 (2.8)	10.0 (2.9)	10.5 (3.4)	10.6 (3.7)	10.4 (3.0)
Lymphomas	9.6 (3.4)	9.8 (3.3)	9.4 (3.4)	9.2 (3.2)*	9.8 (3.1)	8.5 (3.2)#	10.0 (3.4)	9.8 (3.6)	10.2 (3.3)
Fibrosarcoma	11.1 (3.4)	10.4 (2.3)	11.6 (4.0)	10.2 (2.3)*	9.8 (1.6)	10.8 (2.9)	11.5 (3.8)	10.9 (2.6)	11.9 (4.4)
Osteosarcomas	9.0 (3.3)	9.1 (3.0)	8.8 (3.6)	9.1 (3.3)	9.5 (2.9)	8.8 (3.7)	8.1 (3.2)	7.2 (3.4)	9.3 (3.0)

* difference on the mean age between species (total dog versus total cat), $p<0.05$. # difference on mean age between female versus male intraspecie, $p<0.05$. & difference on mean age between sex (total female versus total male), $p<0.05$.
